# Therapeutic potential of *Lawsonia inermis* Linn: a comprehensive overview

**DOI:** 10.1007/s00210-023-02735-8

**Published:** 2023-11-27

**Authors:** Gaber El-Saber Batiha, John Oluwafemi Teibo, Hazem M. Shaheen, Benjamin Ayodipupo Babalola, Titilade Kehinde Ayandeyi Teibo, Hayder M. Al-kuraishy, Ali I. Al-Garbeeb, Athanasios Alexiou, Marios Papadakis

**Affiliations:** 1 Department of Pharmacology and Therapeutics, Faculty of Veterinary Medicine, Damanhour University, Damanhour, 22511 AlBeheira Egypt; 2 Department of Biochemistry and Immunology, Ribeirão Preto Medical School, University of São Paulo, Ribeirão Preto, São Paulo, Brazil; 3 Biochemistry Division, Department of Chemistry, Purdue University, West Lafayette, IN 47907 USA; 4 Department of Maternal-Infant and Public Health Nursing, College of Nursing, University of São Paulo, Ribeirão Preto, São Paulo Brazil; 5 Department of Clinical Pharmacology and Therapeutic Medicine, College of Medicine, Almustansiriyiah University, Bagh-Dad, Iraq; 6 Department of Science and Engineering, Novel Global Community Educational Foundation, Hebersham, NSW 2770 Australia; 7 AFNP Med, 1030 Vienna, Austria; 8 Department of Surgery II, University Hospital Witten-Herdecke, University of Witten-Herdecke, Heusnerstrasse 40, 42283 Wuppertal, Germany

**Keywords:** *Lawsonia inermis*, Pharmacological activities, Bioactive components, Drug development, Therapeutic benefits, Pharmacokinetics, Pharmacological side effects

## Abstract

*Lawsonia inermis* Linn, commonly known as henna, is a member of the Lythraceae family and has been found to contain a variety of compounds with both industrial and medicinal applications in its stem, bark, roots, flowers, and seeds. This report provides a comprehensive review of the bioactive components, pharmacological activities, pharmacokinetics, and pharmacological side effects of *Lawsonia inermis*. Relevant materials were gathered from Google Scholar, PubMed, Scopus, and Web of Science and reviewed for important properties and updates about the plant. *Lawsonia inermis* contains a variety of bioactive compounds, including flavonoids, coumarins, triterpenoids, steroids, xanthones, polyphenols, fatty acids, alkaloids, quinones, tannins, leucocyandin, epicatechin, catechin, and quercetin. The plant is been traditionally used to treat numerous conditions, including ulcers, bronchitis, lumbago, hemicrania, leukoderma, scabies, boils, ophthalmic disorders, hair loss, and jaundice. It has also been found to possess a range of pharmacological activities, including antioxidant, anti-inflammatory, analgesic, antiparasitic, hepatoprotective, antifungal, antitumor, wound healing, and hypoglycemic effects. The potential of *Lawsonia inermis* for various biological applications is promising, and further studies are needed to fully explore its therapeutic benefits for various diseases of public health. Concern advances in drug development could enable the characterization of various bioactive constituents and facilitate their development and application for the benefit of humanity.

## Introduction

**Fig. 1:**
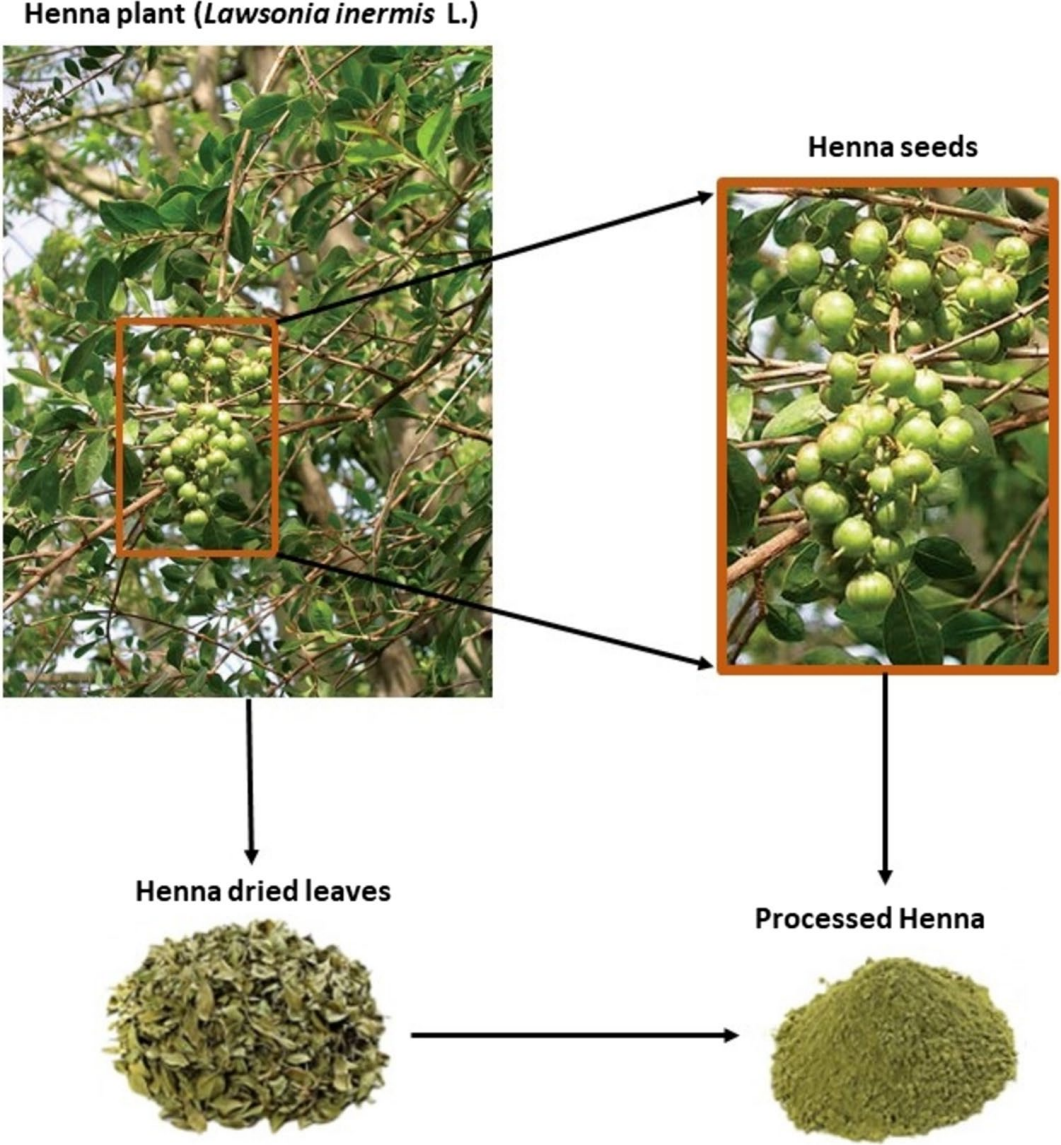
Henna plant and its processing

Many human illness issues, including ulcers, stranguria, cough, bronchitis, lumbago, hemicrania, leukoderma, scabies, boils, ophthalmic disorders, hair loss, and jaundice, are treated with *Lawsonia inermis*, a common herbal treatment (Fig. 1). Henna’s parts are utilized in traditional Chinese medicine as a therapeutic alternative in the treatment of diseases (Yadav et al. 2013). Numerous active substances, including fatty acids, proteins, carbohydrates, and phytochemicals are believed to be present in henna. It serves as a natural source for industrial product synthesis and medication to cure various disorders (Sahu et al. 2012). We examined the medicinal potential of henna in this article as a prospective therapeutic plant and as a unique source of many pharmacologically relevant compounds.

Henna grows to 2.4–5 m and is a heavily branched deciduous, glabrous, occasionally spinescent shrub. It is grown for its dye and as a commercial crop in various Indian states being a hedge plant. Since the henna plant needs little irrigation, it may thrive well in semiarid and arid environments. In areas with limited water resources, it offers a special potential for agriculture. During the months of March and April, its seeds are sowed (Kumar et al. 2005). Three to five times a year, henna is collected, with the best products coming in early July and the worst in February, and multiple harvests of the crop are possible each year (Neeraj et al. 2019).

Every part of the plant has been found to be important, and every of its parts have been reported to be used in the treatment of diseases just like every other plant (Adetobi et al. 2022; Otunba et al. 2022). Use of the leaves was advised by the Indian Ayurvedic Pharmacopoeia for pruritus, bleeding issues, dysuria, and other difficult skin diseases (Khare 2017). The leaf has a bitter taste and is used to treat scabies, boils, ophthalmia, syphilitic sores, amenorrhea, lumbago, headache, and hemicrania. Additionally, it favors hair growth. The leaves are traditionally applied to wounds and ulcers as well as to prevent skin inflammation, and they are used to treat febrile disorders, hemicranias, cephalalgia, leukoderma, and ophthalmia (Warrier et al. 2004). For centuries, henna leaves have been widely used to color textiles, hands, hair, nails, and other body parts (Zumrutdal and Ozaslan 2012). Its bark has been utilized to avoid skin conditions, calculous ailment, spleen and liver enlargement, and jaundice. The essential oil extracted from the blossoms has been utilized for fragrance (Handa et al. 1997). *L. inermis* has also been mentioned to be used as a treatment for malignant ulcers, epilepsy, and jaundice. The flower is used as a refrigerant and to treat insomnia (Abdulmoneim 2007).

*Lawsonia inermis* contains a variety of bioactive compounds, including flavonoids, coumarins, triterpenoids, steroids, xanthones, polyphenols, fatty acids, alkaloids, quinones, tannins, leucocyandin, epicatechin, catechin, and quercetin. These chemical compositions account for the ability of *Lawsonia* to treat ulcers, bronchitis, lumbago, hemicrania, leukoderma, scabies, boils, ophthalmic disorders, hair loss, and jaundice. Some of these phytochemicals have been reported to possess a range of pharmacological activities, including antioxidant, anti-inflammatory, analgesic, antiparasitic, hepatoprotective, antifungal, antitumor, wound healing, and hypoglycemic effects. By utilizing TLC-densitometry, it was found that lawsone was present in *L. inermis* leaves at different concentrations of dried crude drug; however, TLC image analysis found a variation in the concentration of the dried crude drug. And the HPLC results revealed the extracts of parts of the plant (Oda et al. 2018), contained considerable amounts of polyphenols (Babili et al. 2013).

In this review, we reported the various pharmacological activities of *Lawsonia*. Rahiman et al. (2013) examined the antibacterial activities of henna cultivated in vitro and in vivo. Different bacterial strains were used in this study. They discovered that *L. inermis* extracts have antibacterial qualities that are effective against the pathogenic microbes that were being examined. Also, Elansary et al. (2020) discovered considerable antioxidant activity in the leaf extracts of *A. saligna* and *L. inermis*. Compared to the control, they greatly reduced the buildup of oxidative species in all of the cancer cells that were studied. Also, Chaibi et al. (2017) looked into the hexane, chloroform, and methanolic extracts of henna seeds’ anti-inflammatory capabilities. With an IC50 value of 510.23 mg/l, it was confirmed that the methanolic extract exhibited the strongest anti-inflammatory action when compared to all other investigated extracts. Extracts from chloroform and hexane were not especially active, with an IC50 of greater than 100 mg/ml. It was recently reported that the effect of the dye generated from *L. inermis* has an effect on hair growth as published by Bianchi et al. (2020). The presence of phytochemicals in the ethanolic extract has been found to have antiparasitic activity (Barupal et al. 2020). Likewise, its antifungal, hepatoprotective, gastroprotective, hypoglycemic, and wound and burn healing activities have been reported (Hsouna et al. 2016; Hekmatpou et al. 2018; Yassine et al. 2020; Otunba et al. 2021). Chaibi et al. (2017) investigated the anticancer activity of henna seed extracts in hexane, chloroform, and methanol against the colon cancer cell line HTC-116. The strongest cytotoxic impact was demonstrated by chloroform seed extract.

For the pharmacokinetics of *Lawsonia*, it was revealed that it has the ability to eliminate or absorb toxic ions from aqueous solutions. The concentration of the plant extract and the pH of the solution play an important role in influencing the complicated interactions between ions and *Lawsonia inermis*. *Lawsonia inermis* was investigated for the elimination of Zn (II) ions from aqueous solutions (Bhatia and Khan 2014). With 0.2 g/l of biomass, an optimal pH conditions resulted in the highest Zn (II) adsorption. It was found that the pH of the solution played a significant role in influencing the complicated interactions between zinc ions and *Lawsonia inermis*. In this review, we highlighted bentazon’s ability to bind to *Lawsonia inermis* wood-based activated carbon (Abdessalem et al. 2016). The result revealed that the adsorption of bentazon was spontaneous and exothermic between 20 and 40 °C using the derived thermodynamic parameters.

Also, the pharmacological side effect was highlighted in this review. Aqueous *L. inermis* root extract at relatively high concentration caused light-headedness, momentary forgetfulness, and unexpected miscarriage in pregnant female rats (Mudi et al. 2011). Agabna et al. (2014), however, reported safety of *L. inermis* ethanolic seed extracts at doses of 500 and 1000 g/kg in mice. In this study, it is highlighted that after 24 h, neither the acute dose of 500 mg nor the acute dose of 1000 mg/kg resulted in animal death, nor were there any indications of altered feeding, behavior, diarrhea, or fur loss. Also, the blood levels, ions, and macromolecules were not affected. High ethanolic concentrations (80%) of *Lawsonia inermis* were found to have teratogenic effects—a decrease in weight and height of embryos.

Hence, knowledge from this review offers intelligence towards the pharmacological potential of *Lawsonia inermis*. More industrial and research effort and investment on this plant subject are able to contribute immensely towards areas of agro-business, health, and science. In addition, the relevance of this study gives a perspective towards the achievement of Sustainable Development Goal 3 (Babalola et al. 2023). The knowledge and application of the bioactive components, pharmacological activities, pharmacokinetics, and pharmacological side effects as reported in this study will guide the pharmaceutical and research industries to ensure the health of lives and promote the well-being of all, at all ages just as been previously reported (Batiha et al. 2023a, 2023b). Therefore, this present study will also be important to pharmaceutical industries, medical industries, researchers, policymakers, government of nations, and students.

## Botanical description and cultivation

It grows to 2.4–5 m and is a heavily branched, deciduous, glabrous occasionally spinescent shrub. The leaves are frequently mucronulate, base tapering, and have extremely short petioles. They measure 1.3–3.2 by 0.6–1.6 cm. Numerous, smaller than 1.3-cm-long flowers with big terminal pyramidal panicled cymes that are fragrant, white or rose-colored, and have short, slender pedicels (Jallad and Jallad 2008). A persistent calyx supports the 6-mm-diameter, globose, outside-slightly veined, and style-tipped capsules, according to Kirtikar and Basu (2005) and Nadkarni (1982). The crimson, globose, pea-sized seed capsules contain countless tiny, pyramid-shaped, brown-pitted seeds.

It is grown for its dye and as a commercial crop in various Indian states. Since the henna plant needs little irrigation, it may thrive well in semiarid and arid environments. During the months of March and April, seeds are sowed. In August, saplings are planted in the field with a 30 × 30 cm spacing. Stem cuttings can also be used to grow quickly. After the wet season, one or two irrigation and weeding procedures are needed. For the first harvest, the leaves and entire branches are cut/picked in the months of March and April (Kumar et al. 2005).

Three to five times a year, henna may be collected, with the best products coming in early July and the worst in February. To prepare the harvested leaves for further processing, they can be manually or mechanically sorted, graded, and sieved to remove straws, fruits, branches, and dust (Chand et al. 2005). Multiple harvests of the crop are possible each year (Neeraj et al. 2019).

## Ethnobotanical uses of *L. inermis*

Many diseases are treatable using the entire plant (Zumrutdal and Ozaslan 2012). The leaves are traditionally applied to wounds and ulcers as well as to prevent skin inflammation (Nadkarni 1994). Its leaves have also been employed as an expectorant and hematinic to treat febrile disorders, hemicranias, cephalalgia, leukoderma, and ophthalmia (Warrier et al. 2004). Its bark has been utilized to avoid skin conditions, calculous ailment, spleen and liver enlargement, and jaundice. The essential oil extracted from the blossoms has been utilized as fragrance (Handa et al. 1997). According to Warrier et al. (2004), its blossoms are also known to have refrigerant, cardiotonic, febrifuge, soporific, cephalalgia, amentia, and sleeplessness properties.

*L. inermis* is mentioned in both the Charaka Samhita and the Sushruta Samhita as a treatment for malignant ulcers, epilepsy, and jaundice.

## Chemical composition of *L. inermis* Linn

Flavonoids, coumarins, triterpenoids, steroids, and xanthones are among the several phytoconstituents found in *L. inermis*, according to Borade et al. (2011). Ash content was detected in the leaf’s quantitative phytochemical examination (Jain et al. 2010). Both the alcoholic and the aqueous extracts contained detectable levels of biomolecules and phytochemicals. Elansary et al. (2020) identified a number of polyphenols, albeit at speculatively low concentrations, in the methanolic leaf extracts of *L. inermis* using HPLC–DAD.

**Fig. 2:**
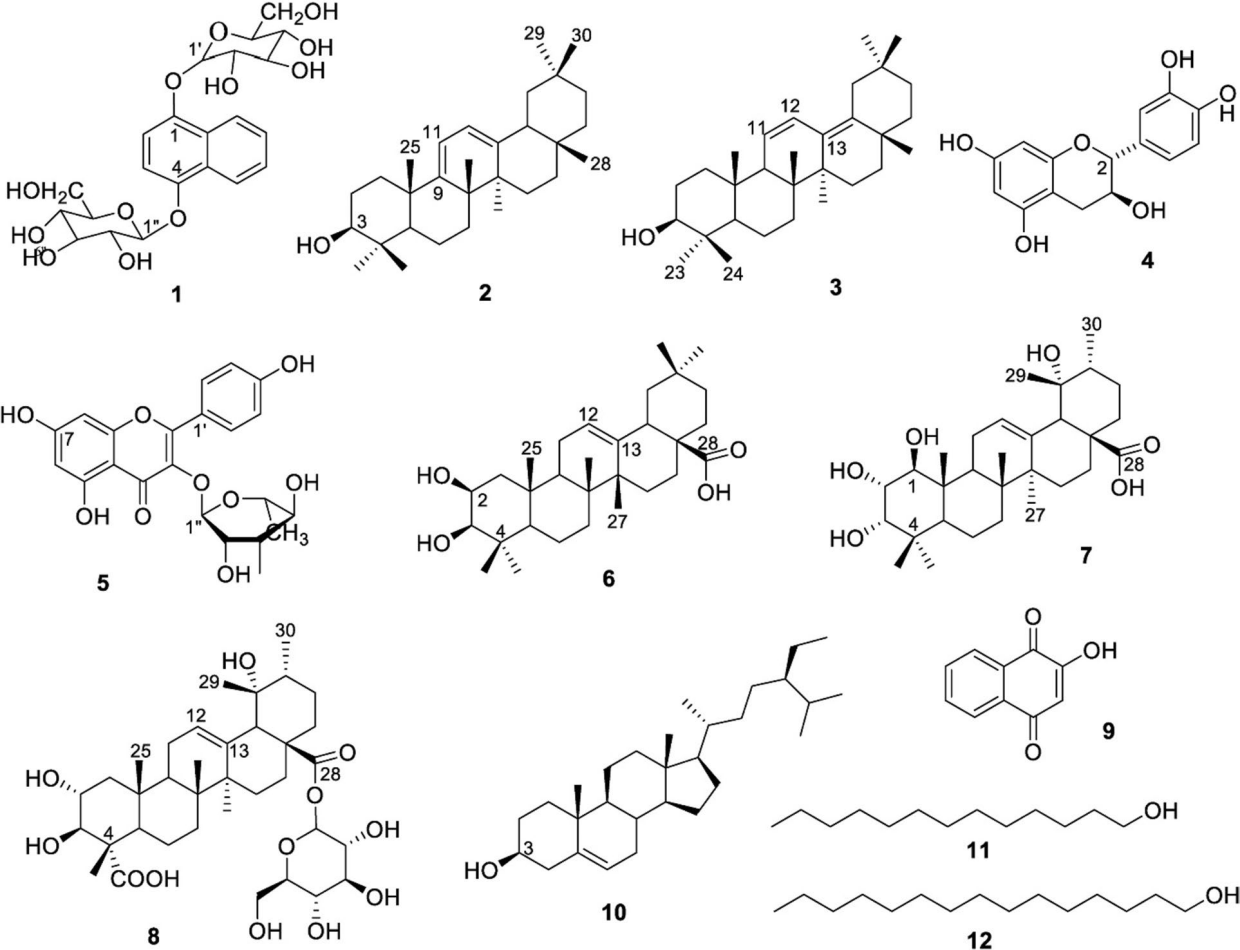
*Lawsonia inermis* L. isolated compound structures 1 through 12. Adapted from Tuan et al. (2022). **1** (rubinaphthin B); **2** (9(11),12-oleanadien-3β-ol; **3** (11,13(18)-oleanadien-3β-ol); **4** (catechin); **5** (afzelin); **6** (augustic acid); **7** (1β, 2α, 3α, 19α-tetrahydroxy-12-ursen-28-oic acid); **8** (suavissimoside R1); **9** (lawsone); **10** (β-sitosterol); **11** (1-tridecanol); **12** (1-pentadecanol)

There have been instances of *L. inermis* containing 2-hydroxy-1,4-naphthoquinone, according to the HPLC results revealed from the extracts of parts of the plant (Oda et al. 2018). According to sources, all parts of the plant contain considerable amounts of polyphenols (Babili et al. 2013). More naphthoquinone derivatives have supposedly been produced from *Lawsonia inermis* leaves (Yang et al. 2017; Hien et al. 2010). Also, Tuan et al. (2022) isolated 12 compounds from *Lawsonia inermis* L. (Fig. 2). They are rubinaphthin B; 9(11),12-oleanadien-3β-ol; 11,13(18)-oleanadien-3β-ol; catechin; afzelin; augustic acid; 1β, 2α, 3α, 19α-tetrahydroxy-12-ursen-28-oic acid; suavissimoside R1; lawsone; β-sitosterol; 1-tridecanol; and 1-pentadecanol.

Flavonoids that are isolated from the plant include apigenin, apigenin-7-glucoside and other derivatives, lutein, kaempferol, quercetin, isoscutellarin, tricine, kaempferin, isoquercitrin, ( −) catechin, 4,′-hydroxyflavanone, 3,7,4′,5′-tetrahydroxy-6-methoxyflavone, 7,3-dimethoxy-6,8-dimethyl flavone, and 3,5′-hydroxyflavone (Iqbal et al. 2017). Research was done on the number of essential oils (EO) in leaves; the majority was composed of oxygenated sesquiterpenes (12.4%) and apocarotenoids (33.6%). Geranyl acetone made up the majority of the EO, accounting for 13.4% of the total. Its comparably low (2.9%) amount of ionene is what gives it its disagreeable smell (Kamal and Jawaid 2010).

The pharmacognostic properties of the leaves were evaluated in accordance with the WHO’s criteria for the quality control procedure for medicinal plant materials (Charoensup et al. 2017). The lawsone’s contents were analyzed using densitometry, image analysis, and thin-layer chromatography (TLC). Mesophyll, parenchyma, an epidermal layer with stomata, and rosette-shaped calcium oxalate crystals could all be seen when the powders were studied under a microscope.

By utilizing TLC-densitometry, it was found that lawsone was present in *L. inermis* leaves at different concentrations of dried crude drug; however, TLC image analysis found a variation in the concentration of the dried crude drug. According to Chaudhary et al. (2010), the plant *L. inermis* contains fatty acids, alkaloids, terpenoids, quinones, coumarins, quinones, tannins, and phenolic compounds. The condensed tannin content of henna leaves was found to be 11.12% (Musa and Gasmelseed 2012). Leucocyandin, epicatechin, catechin, and quercetin were the same as those found in the original samples. 2-Hydroxy-1,4-naphthoquinone was found to be the primary coloring agent in henna. Using ultraviolet light, henna leaf extract was analyzed (UV). Spray drying of an aqueous henna leaf extract using a co-current spray dryer yielded a brown, fine powder with a 33–35% yield. Approximately 70 phenolic compounds were found in various *L. inermes* sections. Lawsone, a coloring component found in naphthoquinones, has been linked to several therapeutic effects (Semwal et al. 2014). Computational studies to understand the interaction and binding affinity of these chemical compounds towards drug targets of pharmacological importance could be conducted (Babalola et al. 2021b).

## Pharmacological activity

**Fig. 3:**
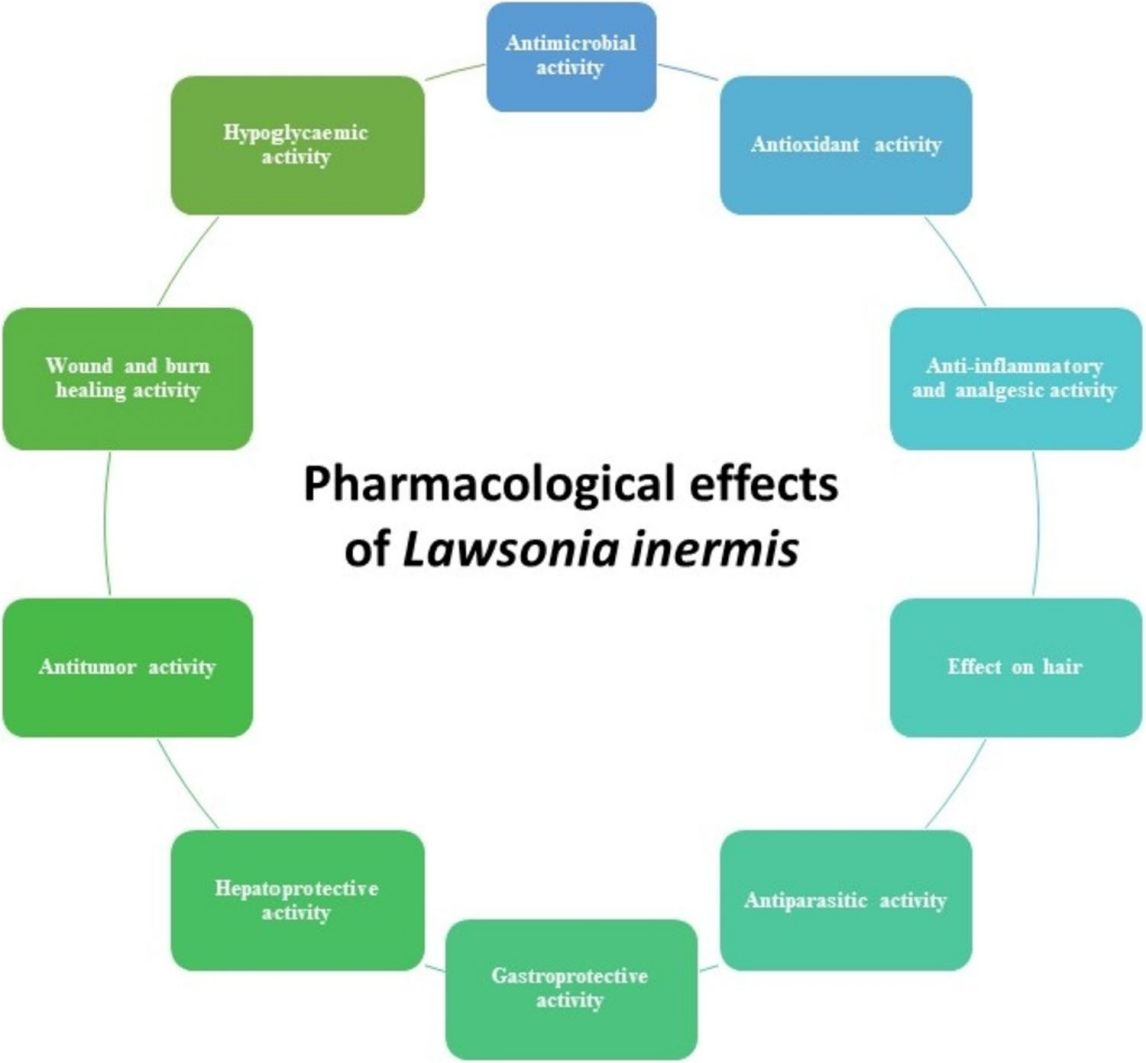
Summary of the pharmacological effects of *Lawsonia inermis*

In this review, the pharmacological effect of *Lawsonia* was discussed (Fig. 3). The pharmacological activities discussed are antimicrobial, antioxidant, anti-inflammatory and analgesic, effect on hair, antiparasitic, gastroprotective, hepatoprotective, antitumor, wound and burn healing, and the hypoglycaemic activities.

## Antimicrobial activity

When combined with plant extract, the antifungal nystatin displayed increased action. The researchers came to the conclusion that every plant extract tested had potential antibacterial action against the examined diseases (Abdelraouf et al. 2011). In comparison to bacteria, the synergistic effect on fungi was stronger and more precise. Al-Mehna and Kadhum (2011) investigated the antibacterial effects of *L. inermis* leaves against *Streptococcus pyogenes*, and they arrived at the conclusion that the activity of the plant is only dependent on the solvent being used. Hence, when combined with plant extract, it shows increased antifungal action. Hence, the plant extract tested had potential antibacterial action against the examined diseases.

*L. inermis* was obtained from diverse locations in Oman, and Habbal et al. (2011) tested its antibacterial effectiveness against a range of microbes. The NCTC 10662 laboratory strain of *Pseudomonas aeruginosa* and eleven brand-new clinical isolates of *P. aeruginosa* were examined for the extracts’ antibacterial activity at varied doses. They discovered that henna samples from the Al Sharqiya Region had antibacterial activity against all isolates and had the highest susceptibility to *P. aeruginosa*. They concluded that Omani henna from the Al Sharqiya Region had the highest activity against *P. aeruginosa* after comparing multiple henna samples from different parts of Oman. Gull et al. (2013) assessed the antibacterial activity of *L. inermis* using seven clinical isolates of bacteria, including gram-negative. However, they came to the conclusion that using *L. inermis* extract as a substitute for antimicrobial medications is crucial.

To corroborate the conventional Ayurvedic belief that this herbal treatment is a Rasayana, Raja et al. (2013) examined the phytochemical and antibacterial properties of *L. inermis* leaf extract. They found that the methanolic leaf extracts of *L. inermis* Linn inhibit microbial growth in a dose-dependent manner. The researchers came to the conclusion that the plant’s antibacterial properties justified its application in the management of bacterial infections. Flavonoids are pharmacologically significant and have potent anti-proliferative activities that are connected to the reduction of cell cycle progression and induction of apoptosis, according to Arun et al. (2010). Additionally, they have cytotoxic, antimicrobial, antioxidant, and chemoprevention action. Considering this, *L. inermis* Linn, the agar well diffusion method was employed to investigate the antibacterial activity of the methanolic extract of L., which was selected because it largely contains flavonoids.

In a 2013 study, Fatimah Abdul Rahiman et al. (2013) examined the antibacterial activities of henna cultivated in vitro and in vivo. Different bacterial strains were used in this study. They discovered that *L. inermis* extracts have antibacterial qualities that are effective against the pathogenic microbes that were being examined. However, they came to the conclusion that there were no appreciable variations in the antibacterial activity of henna leaf in vivo or in vitro. Additionally, the callus created during in vitro development was ineffective against bacteria.

Yusuf (2016) evaluated the antibiotic activity of several *L. inermis* leaf extracts on different bacterial strains using the disc diffusion assay method (*P. mirabilis*, *P. aeruginosa*, *Staphylococcus epidermidis*, and *Enterococcus faecalis*). All of the extracts’ antibacterial qualities were ineffective against the investigated microorganisms.

On comparing the antifungal activity of lawsone ethanolic extract with Listerine mouthwash in diabetics wearing dentures, it was found that patients were further told to use mouthwash twice daily with a volume of 5 ml/rinse for 30 s while CFU was measured, and post-therapeutic samples were then obtained 1 h and 1 week after the administration of the medicine (Sujanamulk et al. 2016). When compared to Listerine mouthwash, they discovered that crude lawsone demonstrated stronger antifungal efficacy. After using the mouthwash for 1 h and 1 week, lawsone showed to be more effective in lowering CFU (*p* < 0.01). The chi-square test was used to analyze subjective symptoms such as taste and smell. Lawsone was found to have a good flavor, and Listerine had a pleasant scent (*p* < 0.01). Listerine mouthwash was observed to cause more burning (Sujanamulk et al. 2016).

The antifungal effects of the aqueous and ethanolic extracts of leaves from *L. inermis* against several strains of *Candida albicans* were studied by Singla et al. (2013). *L. inermis* leaf extracts had a greater effect (20 mm) than aqueous and ethanolic pomegranate peel and seed extracts, and aqueous henna extracts were more effective than ethanolic extracts.

Suleiman and Mohamed (2014) examined the *L. inermis* from Sudan’s antifungal characteristics. Using the maceration (cold procedure), extracts of ethanol and petroleum ether were made from leaf samples. Using an in vitro bioassay, the extracts’ bioactivity to inhibit the growth of the test fungi was identified. They found that both extracts showed antifungal activity against all yeast strains, with the exception of *Pichia fabianii*, which was found to be resistant to both ethanol and ether extracts. Additionally, the results demonstrated the examined fungus’ resistance to antifungal agents. The minimal inhibitory concentrations (MIC) of different concentrations were found to suppress the growth of the tested dermatophytes. They came to the conclusion that Sudanese henna might be employed as an antifungal agent in medical settings.

Nawasrah et al. (2016) looked at the antifungal effects of henna against *C. albicans* adherent to acrylic resin as a viable method for the prevention of denture stomatitis. One hundred eighty acrylic plates were produced using heat-cured acrylic denture resin. Six groups of 30 samples each were formed from the samples. Only polymers and monomers, which were treated in accordance with the customary manufacturer’s instructions, were present in the first group. To treat the remaining five groups, various quantities of Yamani henna powder (Harazi) were added to the polymer. Two alternative approaches were used to evaluate the impact of henna on *C. albicans*: a semi-quantitative slide count and a quantitative analysis based on culture. Samples were raised at 37 °C in artificial saliva containing *C. albicans* (quantitative). They discovered that there was a statistically significant difference in live *Candida* between group B, which contained 1% Yamani henna powder, and the control group. Differences in live *Candida* were also significant when the powder concentration was 7.5% or 10% in comparison to the control group. They came to the conclusion that henna might be applied to acrylic resin dentures to lessen *C. albicans* development on the denture surface, but more research is required to ascertain how this will alter the physical properties of the dentures.

Leaf extracts (chloroform, ethanol, and aqueous extracts) of *Lawsonia elba* were tested in vitro for their antimicrobial activity against seventeen strains of pathogenic bacteria and twelve fungi including seven pathogenic strains (Ferdous et al. 1990). Each of the extract showed good activity against most of the strains tested.

Recently, it was showed that clinically isolated S. oxacillin resistant strains have been exposed to methanol extracts from five different plants, including *Centratherum antherminticum*, *Eucalyptus globulus*, *Lawsonia inermis*, *Punica granatum*, and *Rubia cordifolia*. These extracts have been demonstrated to exhibit anti-bacterial activity. These *S. aureus* methanol extracts have an IC50 of 0.250 to 4.30 mg/ml. Their findings point to the presence of potential antibacterial compounds against *S. aureus* in a variety of medicinal plants, which may help researchers create more robust treatments for infections caused by the multidrug-resistant bacterium (Assiri et al. 2023).

Patent from Parachur and Ravichandran (2017) showed the anti-fungal activities against fungal infections caused by fungal infections, including those caused by (*Candida* sp., *Cryptococcosis* sp., *Aspergillus* sp., *Penicillium* sp., *Tinea* sp., and *Blastomyces* sp.) using the ethyl acetate extracts or active ingredients isolated from ethyl acetate extract of *Lawsonia inermis*.

## Antioxidant activity

Elansary et al. (2020) discovered considerable antioxidant activity in the leaf extracts of *A. saligna* and *L. inermis*. Compared to the control, they greatly reduced the buildup of oxidative species in all of the cancer cells that were studied. Hosein and Zinab (2007) assessed the use of henna leaf extract as a source of free radicals. It was investigated how adding henna leaf extracts affected the stability of soybean oil, and the total amount of phenolic compounds in the extract was identified using a spectrophotometric approach. The total phenolic components of the extracts were improved, and the extraction periods were shortened, according to Hosein and Zinab (2007) research. They came to conclude that this content and the antioxidant activity of the extract are significantly impacted by the extraction technique.

The Folin–Ciocalteau method was used to assess the polyphenolic content, and DPPH radical scavenging activities (RSA) were used to analyze the antioxidant capabilities of the leaf extracts (Zohourian et al. 2011). They discovered that the mild temperature range of 100–120 °C produced the optimum values of RSA. Better outcomes were obtained with power management of the microwaves during brief irradiation times than with temperature control alone. They came to the conclusion that microwave extraction was more efficient in removing polar components, which typically have higher antioxidant activity. Hence, may have a better outcome when obtained with the power management of the microwaves.

The goal of Asma Elaguel et al. (2019) study was to maximize the yield of essential oil extraction from leaves of *L. inermis* while also identifying its chemical makeup, antioxidant activities, and antiproliferative and lipid peroxidation effects. They discovered that 6.8 g/100 g was the ideal extraction yield. Additionally, Elaguel et al. (2019) noted a marked decline in ERO production in the Raji cell line. Henna essential oil’s anti-tumor properties revealed an intriguing cytotoxic impact.

On rat excision and incision wound models, Sakarkar et al. (2004) examined the wound healing ability of several henna leaf extracts (*L. inermis* Linn). They saw significant healing responses in both wound models following oral ingestion and topical application of ethanol extracts of henna leaves with lawsone. Additionally, they discovered that applying isolated lawsone or an ethanol extract topically was more beneficial than doing so orally. They came to the conclusion that ethanolic extract may be successfully prepared for wound healing activities through topical application.

In the liver, the effects of ethanolic extract of fresh *Lawsonia inermis* leaves on drug-metabolizing enzymes were examined (Dasgupta et al. 2003). In terms of the antioxidant enzymes, both dose levels of the examined doses significantly increased the hepatic glutathione reductase, superoxide dismutase, and catalase activity. Both of the tumor model systems under investigation showed a significant decrease in tumor burden. Both of the doses utilized in the experiment lowered the incidence of tumors in both model systems (Dasgupta et al. 2003).

The primary pharmacological effects of henna and the separated chemicals like lawsone and coumarin were reviewed by Wagini et al. (2014a). Strong antioxidant henna has been shown to reduce or stop the production of free radicals. It is regarded as a secure herbal medication with negligible and infrequent negative effects.

Dhouafli et al. (2017) announced the discovery of a novel chemical, glucopyranoside, which was isolated from lichen *L. inermis* and possessed potent antioxidant properties. Chaibi et al. (2017) have reported on the *L. inermis* methanolic extract’s antioxidant properties utilizing the DPPH free radical scavenging experiment *L. inermis* has shown significant antioxidant activity in their investigation (IC_50_ = 17.0689 g/ml).

Jacob et al. (2011) showed in every assay that the ethanolic extract showed higher antioxidant activity when compared with other extracts. According to reports, the aqueous extract’s activity was higher than petroleum ether and dichloromethane, but less potent than ethanol extract. Higher phenolics were observed compared to the different extracts in this study.

## Anti-inflammatory and analgesic activity

*L. inermis* leaves, which are used in conventional medicine, contain anti-inflammatory qualities, according to Nadkarni (1982). Using chromatographic and spectroscopic techniques, a pure molecule was isolated and identified as 2-hydroxy-1,4naphthaquinone (lawsone), which has significant anti-inflammatory, and analgesic properties, according to Ali et al. (1995). Chaibi et al. (2017) looked into the hexane, chloroform, and methanolic extracts of henna seeds’ anti-inflammatory capabilities. With an IC50 value of 510.23 mg/l, it appeared that the methanolic extract exhibited the strongest anti-inflammatory action when compared to all other investigated extracts. Extracts from chloroform and hexane were not especially active, with an IC50 of greater than 100 mg/ml.

In osteoarthritic rats, the effects of *L. inermis* leaves and the aqueous extract of *R. communis* leaves were studied by Ziaei et al. (2016). Knee osteoarthritis was brought on by monosodium iodoacetate. Following a 3-day injection period, Ziaei et al. (2016) found that the extract mixture significantly reduced the knee joint breadth and volume of the injected paws and improved gait analysis footprints.

Manivannan and Aeganathan (2016) evaluated the analgesic effectiveness of various *L. inermis* leaf extracts in mice using hot-plate and acetic acid-induced writhing techniques. They discovered that every extract displayed a significant analgesic impact in a dose-dependent manner in some pain models. Additionally, Sultana and Khosru (2011) examined the analgesic properties of an ethanol extract of *Lawsonia inermis* leaf using mice whose writhing was produced by acetic acid. They found that there was no discernible reflex inhibition caused by diclofenac sodium.

## Effect on hair

The evaluation of the effect of the dye generated from *L. inermis* on the hair was published by Bianchi et al. (2020). Scanning electron microscopy was used to assess dyed hairs. They were then evaluated qualitatively and quantitatively in comparison to undyed hairs. After using henna, Bianchi et al. (2020) demonstrated that the genotype and the health of the hair prior to dyeing may have an impact on the type and extent of morphological alterations that occur on colored hairs.

## Antiparasitic activity

The anthelmintic activity of *Lawsonia inermis*’ ethanolic and methanolic extracts was assessed by Sarojini et al. (2012). The extracts were discovered to have stronger anthelmintic activity than the positive control and to have an anthelmintic activity that was dose-dependent. In order to link the results of the phytochemical screening with their anthelmintic action, the extracts’ phytochemical composition was also assessed. They came to the conclusion that while methanolic extract was likely to be useful due to the presence of phytochemicals, the ethanolic extract was strong as an anthelmintic agent (Barupal et al. 2020).

In a thorough extraction, many chemical compositions were found to be present (Babili et al. 2013). There were flavonoids (16.2–85.6 g/kg for quercetin), anthocyanins (0.75–5.48 mg/kg), tannins (31.3–477.9 g/kg for catechin), and polyphenols (71.7–129.6 g/kg for gallic acid). In addition, they gave the outcomes of checks for antimalarial activity, ABTS/DPPH assay, and MCF7 human breast cancer cell activity. They found that the extract with the strongest antioxidant activity was produced using ethanol.

Bakhshi et al. (2014) looked into the larvicidal effectiveness of *L. inermis* against the malaria vector *Anopheles stephensi*. The tested extract was more damaging to larval stages I and II at particular doses. It was shown that stages III and IV of the larvae had a similar outcome. For larval stages, the fatal dose values varied with concentration.

Using 70% methanol as the extractant, Ismail et al. (2016) investigated the in vitro anti-*Strongyloides* action of the stem of *L. inermis*. Different extract concentrations (1, 10, and 100 mg/ml) were incubated with the larvae and free-living females for various incubation times (24, 48, 72, and 96 h). They discovered that, in contrast to flubendazole (100 mg/ml), which had no effect on the parasite’s cuticular surface, *L. inermis* at a dosage of 10 mg/ml for 24 h caused fissures and depression.

The antileishmanial activity of methanolic extracts of *L. inermis* (0.07, 0.15, 0.31, 0.62, 1.25, 2.5, 5, 10 mg/ml) on *Leishmania* was examined by Motazedian et al. (2017) using the MTT test. A methanolic extract of *L. inermis* with an IC50 of 1.25 mg/ml inhibited promastigote forms of L. major in vitro after 72 h of incubation. However, the in vivo investigation revealed that the disease status tends to improve with the treatment. Tadesse and Mirutse (2009) concluded after completing in vitro testing that *Lawsonia inermis* extracts have no antihelminthic effect.

Anna et al. (2016) examined the anticoccidial properties of a 90% ethanolic extract of leaves from *L. inermis* against caecal coccidiosis in broilers. In comparison to salinomycin, the 300-ppm dose of *Lawsonia inermis* leaf extract as a feed supplement had good anticoccidial activity and considerably decreased lesions and mortality.

## Gastroprotective activity

In albino rats, Goswami et al. (2011) investigated the gastroprotective effects of *L. inermis*. The study found that *L. inermis* reduced stomach volume, acidity, and ulcer index. Chloroform extract had the strongest gastroprotective effect when compared to alcoholic and aqueous leaf extracts.

Peptic ulcer disease (PUD) is one of the main illnesses and deaths around the globe. Mucosal lining erosions in the stomach and duodenum are its hallmarks (Majeed et al. 2015). Histamine receptor blockers and proton pump inhibitors are the two most often used drugs to treat peptic ulcers. NSAIDs, however, have been associated with harmful side effects. As a result, interest in herbal ulcer treatments is currently growing. The body’s defences against peptic ulcers are boosted by a variety of phytoconstituents, which are substances found in plants.

Basipogu and Syed (2015) evaluated the pharmacological effect of *L. inermis* leaf, folk, and ethnomedicine used to heal stomach ulcers. The methanolic extract from *L. inermis* leaves demonstrated remarkable protection against tissue ulceration. As a result, it supports its historical use as an ulcer remedy in India.

Henna contains cooling and protecting qualities that help prevent decubitus ulcers in intensive care units, claim Davood Hekmatpou et al. (2018). A randomized clinical trial included 80 patients receiving care in intensive care units at hospitals. By random allocation, the patients were split into the control and intervention groups (*n* = 40). Henna was applied to the patient’s sacrum in the intervention group along with the standard prophylactic treatments for decubitus ulcers. Six individuals in the control group had developed decubitus ulcers, which were found to have occurred at the trial’s conclusion. This variation is statistically noteworthy.

## Hepatoprotective activity

Sanni et al. (2010) examined the hepato-protective effects of an aqueous leaf extract of *L. inermis* on carbon tetrachloride–induced liver damage in Swiss albino mice. They did this by measuring the blood levels of alanine aminotransferase (ALT) and aspartate aminotransferase (AST). They found that the extract significantly reduced the serum levels of AST and ALT without being dosage dependent. They discovered that *L. inermis* aqueous leaf extracts exhibit hepatoprotective qualities when taken in the right dosage.

Mohamed et al. (2016) investigated the hepatoprotective properties of a methanolic extract from leaves of *L. inermis* on carbon tetrachloride–induced liver injury in rats (CCl4). The tested animals were given oral doses of 100 mg/kg and 200 mg/kg of the extract in order to assess the effects of the *L. inermis* leaf methanolic extract on serum levels of hepatotoxicity parameters and histopathological liver sections examination. They found that the two doses of the plant extract displayed dose-dependent hepatoprotective impact. They arrived at the conclusion that this plant material’s antioxidant properties might have a hepatoprotective effect.

Kumar et al. (2017) examined the in vitro antioxidant and in vivo hepatoprotective properties of the butanolic fraction of *L. inermis* leaves in order to shield the livers of male Wistar rats from 2-acetylaminofluorene (2-AAF)–induced hepatic damage. The butanoic fraction effectively scavenged hydroxyl radicals in the deoxyribose breakdown experiment. It also dramatically raised the reducing potential in the FRAP experiment and stopped lipid peroxidation. Different amounts of the butanoic fraction significantly decreased the SGOT, SGPT, ALP, and lipid peroxidation brought on by 2-AAF therapy, demonstrating considerable hepatoprotective effects. The liver’s native architecture was also restored, as evidenced by the hepatoprotective effect.

Lawsone is a significant bioactive naphthoquinone found in *L. inermis* and was examined by Darvin et al. (2018) in HepG2 cells subjected to RIF-INH and RIF-INH caused hepatotoxicity in Wistar rats. RIF-INH administration decreased the viability of HepG2 cells, and lawsone treatment considerably restored that vitality, even at a lower dose (7.5 M). Treatment with lawsone also significantly decreased MDA levels and transaminase leakage. The serum transaminases and bilirubin levels of the rats given RIF-INH considerably decreased after receiving lawsone treatment, and the albumin to globulin ratio improved.

In order to protect rat liver from carbon tetrachloride (CCl4)–induced oxidative stress, Hsouna et al. (2016) studied the antioxidant and hepatoprotective characteristics of several fractions derived from the fruits of *L. inermis*. Numerous *L. inermis* fruit fractions had significant antioxidant activity.

## Antitumor activity

Priya et al. (2011) conducted an experimental study to examine the possible anticancer effects of an ethanol extract of the root of *L. inermis* against mice with Dalton’s lymphoma ascites (DLA). The ethanolic root extract reportedly decreased the RBC count, hemoglobin content, and monocytes while reversing the rise in WBC, platelets, and lymphocytes. *L. inermis* root extract adversely impacted the pathophysiological marker enzyme, lipid profile, and antioxidant activity. Histological examination of DLA-infected mice revealed loss of liver hepatocytes and renal architecture. However, therapy with *L. inermis* extract improves kidney and liver function in addition to rearranging the mice’s more or less normal architecture.

According to Ozaslan et al. (2009), *L. inermis* can induce apoptosis in cancer cells by either raising intracellular levels of free radicals and H_2_O_2_ due to oxidative activity or by lowering intracellular levels of H + ions. The methanolic leaf extracts and newly found polyphenols from *L. inermis* displayed antiproliferative and cytotoxic effects against cancer cells because necrotic cells amass during apoptotic phases (Elansary et al. 2020).

In mice with hepatocellular carcinoma brought on by nitrosamine, Hamid et al. (2015) investigated the anticancer effects of *L. inermis* and octreotide total methanolic extract. They discovered that *Lawsonia inermis* methanolic extract and octreotide treatment had powerful chemopreventive effects because of their capacity to reduce oxidative stress and desensitize cellular growth receptors to SST. Chaibi et al. (2017) investigated the anticancer activity of henna seed extracts in hexane, chloroform, and methanol against the colon cancer cell line HTC-116. The strongest cytotoxic impact was demonstrated by chloroform seed extract. More studies on the effect of *Lawsonia* on cancer immune evasion can be conducted to understand its mechanism of action (Babalola et al. 2021a). Also, this can be conducted on other hallmarks of cancer.

## Wound and burn healing activity

Nayak et al. (2007) evaluated the effects of an ethanol extract of *L. inermis* on the capacity of rats to heal their wounds. In contrast to wound models, excision wound models required topical treatments. Animals treated with extracts had wound areas reduced by 71% as compared to controls, who had wound areas reduced by 58%. Based on histological findings, enhanced skin-breaking strength, hydroxyproline, and wound contraction, *Lawsonia inermis* may be utilized to treat wound healing (Nayak et al. 2007).

Henna leaf extracts have been shown by Muhammad and Muhammad (2005) to have the ability to stop the growth of the bacteria that cause infected burn wounds. This study thus supports the use of henna in the treatment of burn wounds and associated infections. Researchers looked into how the main invaders of burn wounds were impacted by *L. inermis* leaf extracts in water and chloroform. Sakarkar et al. (2004) examined the potential of different *L. inermis* leaf extracts and lawsone to stimulate wound healing. They found that the topical and oral administration of ethanol extracts of henna leaves and lawsone significantly affected both wound models.

A daily application of an ointment made with powder from LI leaves on Wistar rats’ wounds was tested by Yassine et al. (2020). For this study, twenty Wistar female rats were employed. Skin was removed from each animal’s dorsal neck to create an excisional wound model. Then, in the test group, wounds were treated by applying LI ointment daily, which was made by combining petroleum jelly and leaf powder, and petroleum jelly alone in the control group. The healed excised wound was then histologically examined to assess the wound-healing process. This was accomplished by calculating the wound index, length of epithelialization, and wound contraction % over the course of 24 days. They found that therapy with LI had excellent wound healing activity because it increased the percentage of wound contraction and lowered the period of epithelialization and wound index compared to the control. We can infer that LI leaves may be utilized as a substance to aid in the healing of wounds.

Recent studies from Skowrońska and Bazylko (2023) where severe burns have been treated with the herbal ointment fundermol, which comprises *Lawsonia inermis* and beeswax. The preparation’s precise chemical makeup is not known. In a clinical trial, it was compared to 1% SSD cream in the treatment of second-degree burns. The trial included 50 patients who arrived at the clinic with burns that covered 1 to 10% of their entire body surface and were caused by contact with a heater or hot liquid within 6 h. Patients were divided into two equal groups and given either 1% SSD cream or fundermol ointment once daily.

## Hypoglycaemic activity

In a 2017 study, Antika (2017) investigated how ethanol-extracted henna leaves affected blood sugar levels and superoxide enzyme activity. Wistar mice strains were used in the posttest-only control group design study. Antika (2017) discovered a substantial decline in blood glucose levels when compared to the control group. Although the enzyme activity of superoxide dismutase rose, it was not statistically significant. Although mice’s blood glucose levels were dramatically lowered by the 400 mg/kg ethanol extract, there was no discernible difference in the rise in SOD activity.

In normal and diabetic rats, Choubey (2010) investigated the hypoglycemic and antihyperglycemic effects of ethanolic extract. Streptozotocin was administered intraperitoneally once, and albino rats developed diabetes as a result. *L. inermis* ethanolic extract was administered orally for 28 days at doses of 150, 300, and 500 mg/kg of body weight while blood glucose levels were tracked. The blood glucose levels in diabetic rats significantly increased. After being given an oral dose of an ethanolic extract of *Lawsonia inermis*, diabetic rats had significantly reduced blood sugar levels. The effect of ethanolic extract at 500 mg/kg was shown to be superior to glibenclamide (10 mg/kg body weight). These results suggest that the ethanolic extract possesses strong anti-diabetic abilities.

*Lawsonia inermis* significantly reduced blood sugar and cholesterol levels in mice with diabetes caused by alloxan. The extract normalizes blood levels of triglycerides, cholesterol, and glucose, claim Arayne et al. (2007).

In mice with alloxan-induced diabetes, Abdillah et al. (2008) determined the effects of a 70% ethanol extract from *L. inermis* leaves on blood sugar, total cholesterol, and triglycerides. Findings show that the glucose levels decreased 14 days after eating the extract at a rate of 0.8 g/kg body weight. Total cholesterol decreased, and so did the triglyceride content.

## Pharmacokinetics

Bentazon’s ability to bind to *Lawsonia inermis* wood-based activated carbon (LWAC) was investigated (Abdessalem et al. 2016). The effects of various reaction parameters on bentazon adsorption were investigated in batch process mode. It was found that the pseudo-second-order kinetic model and the adsorption kinetic model fit each other. The spontaneous and exothermic adsorption of bentazon onto LWAC was proved to be feasible between 20 and 40 °C using the derived thermodynamic parameters Δ*G*, Δ*H*, and Δ*S*.

Bhatia and Khan (2014) investigated *Lawsonia inermis* for the elimination of Zn (II) ions from aqueous solutions. Investigations were conducted into the effects of a number of operational parameters. With 0.2 g/l of biomass, optimal pH conditions resulted in the highest Zn (II) adsorption. It was found that the pH of the solution played a significant role in influencing the complicated interactions between zinc ions and *Lawsonia inermis*. In 60 min, the biosorption equilibrium was attained. Hence, it can be inferred that *L. inermis* has the ability to eliminate or absorb toxic ions from aqueous solutions. Hence, from this study, it can be seen that the concentration of the plant extract and the pH of the solution will play an important role in influencing the complicated interactions between ions and *Lawsonia inermis*.

Hassan et al. (2014) published a new simple and precise kinetic spectrophotometric method for the measurement of cefadroxil in both its pure form and pharmaceutical formulations. The proposed method was based on the production of a yellow product (max = 410 nm) from an aqueous solution containing cefadroxil, henna extract, sodium hydroxide, and potassium permanganate. The experimental configuration is ideal. Cefadroxil levels in pharmaceutical dosage forms have been evaluated with success using the suggested method.

## Toxicity and pharmacological side effect

The aqueous extract of *L. inermis* was found to be safe in mice up to 2 g/kg bw. There were no fatalities or poisoning signs after 24 h (Darvin et al. 2018). *L. inermis* aqueous root extract toxicity was examined in rats at dosages variation. Light-headedness, momentary forgetfulness, and unexpected miscarriage in pregnant females’ rats were observed. Rats receiving daily doses average dose remained active and healthy. No mortality was recorded across all dosages. The results after administering the extract intraperitoneally at various concentrations revealed delayed toxicity (Mudi et al. 2011).

Agabna et al. (2014) investigated the safety of *L. inermis* ethanolic seed extracts at doses of 500 and 1000 g/kg in mice. After 24 h, neither the acute dose of 500 mg nor the acute dose of 1000 mg/kg resulted in animal death, nor were there any indications of altered feeding, behavior, diarrhea, or fur loss. The number of WBC, RBC, and platelets was barely impacted. The levels of serum Na, K, creatinine, and urea did not differ from the norm. The continuous administration of henna extract had no effect on the liver enzymes, proteins, blood sugar, or lipid profile. However, the high-dose group experienced a minor but significant increase in AST. An autopsy revealed no evidence of poisoning.

The teratogenic effects of 80% ethanol extract at specific concentrations of *Lawsonia inermis*’ aerial organs were examined in mice. Jafarzadeh et al. (2015) conducted this study. The extract at both doses considerably decreased the weight and height of the embryos as compared to the control. At these concentrations, there was no appreciable difference in the weight and height of the embryos. Both *L. inermis*–treated groups showed various frequencies of skeletal abnormalities, such as rib and parietal bone deformities, anencephaly, and exencephaly of embryos. Hence, it can be inferred that the ethanolic extract of the plant possesses teratogenic effects; as seen from the study, it significantly decreased the weight and height of the embryos as compared to the control.

Khine (2017) reported a case of acute renal damage in a 34-year-old male from Yangon, Myanmar, who had G6PD deficiency and had consumed an herbal medicine made from boiling henna leaves. He developed hemoglobinuria, and hemodialysis was performed five times on him. Within 7 weeks, his condition got better, and he recovered completely. Hence, its role can be seen in glucose metabolism and in the management of diabetes.

## In vivo studies on *L. inermis*

Recent in vivo studies, demonstrate the potential of *L. inermis*. Studies from Tauheed et al. (2016) at 250 mg/kg, the extract considerably (*p* = 0.05) decreased parasitaemia levels. At week 2, PCV was greater (*p* > 0.05) in extract-treated groups than in group V, although it was considerably higher (*p* = 0.05) in group II. Compared to groups IV and V, rats in group II showed considerably reduced EOF and MDA values. As a result, the leaf of *L. inermis* not only has an antitrypanosomal effect against *T. congolense* in vivo rats, but also has a reducing effect on trypanosomosis pathology. This action is likely caused by protecting the erythrocyte membrane from oxidative damage caused by trypanosomes to the erythrocytes.

Also, Wagini et al. (2014b) showed that with the aid of 15 naturally infected goats, an in vivo study was conducted. Animals in the first, second, and third groups were given henna paste, aqueous extract, and ethanolic extract, respectively; the fourth group received clotrimazole as a positive control, and the fifth group, the negative control, received neither henna nor clotrimazole. Henna paste exhibited the highest efficacy against all the forms of ringworm evaluated when compared to the other therapies, according to the comparison of the treatments. At 30 days following therapy, the lesion vanished and the hair fully recovered. The group of rats treated with aqueous extract, ethanol extract, and clotrimazole showed remarkably identical results across all criteria. Significant variations were only seen between those that received treatment.

## Conclusion and perspective

*Lawsonia* is a pharmacologically important plant, and this study shows that every part of the plant has been used as substances for traditional medicine. It serves as a natural source for industrial product synthesis as well as medications to cure various disorders. From this review, we have some recommendations that can help the scientific and pharmaceutical community understand further benefits of the plant. First, we recommend that more studies on the potential of *Lawsonia inermis* as a therapeutic plant and a source of pharmacologically relevant compounds should be done, as well as more studies on the agricultural potential of henna. We recommend that future studies investigate further the potential pharmacological properties with specific reference to both in vitro and in vivo studies. This will help establish the full range of therapeutic benefits that can be derived from the plant. More specifically, we speculate that exploring the potential of henna as a natural source for industrial product synthesis and development of new medications to cure various disorders will engender the production of safer drugs with less toxicity compared to the synthetic, an action which should be encouraged. We further encourage the development of new formulations of henna extracts for topical and internal use to improve the bioavailability and efficacy of the bioactive compounds. Also, clinical trials to evaluate the safety and efficacy of henna extracts for the treatment of various human illnesses can be considered. Lastly, we recommend that the investigation of the mechanism of action of the bioactive compounds in henna to understand their therapeutic potential and develop new drug targets be studied in details.

## Data Availability

Data sharing not applicable to this article as no datasets were generated or analyzed during the current study.
