# Correction: Therapeutic potential of *Lawsonia inermis* Linn: a comprehensive overview

**DOI:** 10.1007/s00210-023-02932-5

**Published:** 2024-01-02

**Authors:** Gaber El‑Saber Batiha, John Oluwafemi Teibo, Hazem M. Shaheen, Benjamin Ayodipupo Babalola, Titilade Kehinde Ayandeyi Teibo, Hayder M. Al‑kuraishy, Ali I. Al‑Garbeeb, Athanasios Alexiou, Marios Papadakis

**Affiliations:** 1 Department of Pharmacology and Therapeutics, Faculty of Veterinary Medicine, Damanhour University, Damanhour, 22511 AlBeheira Egypt; 2 Department of Biochemistry and Immunology, Ribeirão Preto Medical School, University of São Paulo, Ribeirão Preto, São Paulo Brazil; 3 Biochemistry Division, Department of Chemistry, Purdue University, West Lafayette, IN 47907 USA; 4 Department of Maternal‑Infant and Public Health Nursing, College of Nursing, University of São Paulo, Ribeirão Preto, São Paulo Brazil; 5 Department of Clinical Pharmacology and Therapeutic Medicine, College of Medicine, Almustansiriyiah University, Bagh‑Dad, Iraq; 6 Department of Science and Engineering, Novel Global Community Educational Foundation, Hebersham, NSW 2770 Australia; 7 AFNP Med, 1030 Vienna, Austria; 8 Department of Surgery II, University Hospital Witten-Herdecke, University of Witten-Herdecke, Heusnerstrasse 40, 42283 Wuppertal, Germany

**Correction: Naunyn–Schmiedeberg's Archives of Pharmacology**

10.1007/s00210-023-02735-8

In the published version of the original paper, the name of the fourth author has been misspelt.

The name Benjamin Ayodupupo Babalola should be corrected as "Benjamin Ayodipupo Babalola".

The original article has been corrected.

